# Detecting damaged buildings using real-time crowdsourced images and transfer learning

**DOI:** 10.1038/s41598-022-12965-0

**Published:** 2022-05-27

**Authors:** Gaurav Chachra, Qingkai Kong, Jim Huang, Srujay Korlakunta, Jennifer Grannen, Alexander Robson, Richard M. Allen

**Affiliations:** 1 University of California, Berkeley, Berkeley, USA; 2 Lawrence Livermore National Laboratory, Livermore, USA; 3 Berkeley Seismological Laboratory, University of California, Berkeley, Berkeley, USA; 4 AT&T, Dallas, USA

**Keywords:** Seismology, Natural hazards

## Abstract

After significant earthquakes, we can see images posted on social media platforms by individuals and media agencies owing to the mass usage of smartphones these days. These images can be utilized to provide information about the shaking damage in the earthquake region both to the public and research community, and potentially to guide rescue work. This paper presents an automated way to extract the damaged buildings images after earthquakes from social media platforms such as Twitter and thus identify the particular user posts containing such images. Using transfer learning and ~ 6500 manually labelled images, we trained a deep learning model to recognize images with damaged buildings in the scene. The trained model achieved good performance when tested on newly acquired images of earthquakes at different locations and when ran in near real-time on Twitter feed after the 2020 M7.0 earthquake in Turkey. Furthermore, to better understand how the model makes decisions, we also implemented the Grad-CAM method to visualize the important regions on the images that facilitate the decision.

## Introduction

Large earthquakes near populated areas leave unforgettable testimony of their intimidating powers: collapsed buildings, offset roads, disrupted lives, etc. With the help of current technology, images of this damage can be captured soon after the earthquake and uploaded online so that people in other parts of the world can easily browse through these images and videos to obtain a sense of the consequences of the disaster. Such images or videos taken by individuals or news media often contain important information about the damage, such as damaged buildings, bridges, roads and other infrastructures^1,2^.

There are existing efforts to extract useful information from these crowdsourcing images to better understand natural disasters. Nguyen et al.^3^ used a transfer learning approach, more specifically, domain-specific fine-tuning Convolution Neural Network (CNN) to estimate the damage severity in the images on social media platforms after a disaster. Alam et al.^4^ provided a social media image processing pipeline to combine human and machine intelligence to extract information from images as a situational awareness task during a crisis event, such as earthquakes or hurricanes. They used the pre-trained VGG-16 model with fine-tuning to filter the relevant images from the collected images on Twitter. After the removal of the duplicated images and crowdsourcing efforts to label the images, they fed these images into an image classifier to identify damaged structures, injured people and rescue efforts. Using CNN and transfer learning, Hassan et al.^5^ analyzed the visual sentiment from disaster images in social media, which can estimate sentiments such as joy, fear, anger, sadness etc. from the images related to disasters. Hao and Wang^6^ used two modules in their pipeline to analyze multimodal social media data, such as text and images. The designed image analysis submodule used five machine learning classifiers (support vector machine, logistic regression, artificial neural network etc.) to estimate whether a hazard was present in the image, hazard type, hazard severity etc. Combining the text analysis they can provide a good description of disaster damage both from text and image. There are many other research efforts in this area, and many are building a pipeline for multi-hazards or multimodal datasets, which differ from the research that we demonstrate below.

In this research, we use a transfer learning based approach to select images from social media platforms that contain damaged buildings, so they can be used in an application we developed in the earthquake science domain. The motivation of this work came from the earthquake crowdsourcing application MyShake^7–11^, which recently started to allow users to upload felt reports after an earthquake and display the felt region to the users^12,13^. These reports are short surveys that the users can complete with pre-defined options for the felt experience and observation of damaged structures. With these felt reports, there are also plans to enable image uploading from users after an earthquake, which may include images of damaged buildings, roads, and bridges in the area. These images are not only useful to the MyShake users to learn where there is damage around them, but also can provide a valuable dataset for the research community and emergency services communities, especially coupled with locations (felt reports in MyShake have the user location). Potential applications can be developed from this information including the severity of the damage, better maps of shaking distribution and damage, and prioritization guidance for the rescue community. However, certain users may upload images that are not related to damage. Therefore, we must build a machine learning based approach to select only the images containing damaged buildings.

The approach and results described below include a model we built by using transfer learning with a pre-trained model VGG19^14^, which is a model trained on the ImageNet dataset^15^. Transfer learning has been shown to be a very effective way to build a model in many different fields^16^ when obtaining sufficiently large target training datasets is a challenge. Instead, by fine tuning the last few layers of a well pre-trained model that was trained using similar large datasets, good performance can be achieved even with relatively small datasets. Using 6556 manually labelled images downloaded from Twitter and Getty Images, we fine tuned the last block of the VGG19 model, and trained the model to recognize which images contain damaged buildings in the scene. To test the performance of the built model, we downloaded a new test dataset (Test Dataset 1) from Twitter during the period 2020-01-01 to 2020-09-30 with 20,804 images. The results from these tests are good; the damaged building class recall is 80.5% with a precision of 77.4%; non-damage class recall is 99.6% with a precision of 99.6%. In addition to evaluating the model using the Test Dataset 1, we also ran the model in near real-time for 30 hours on Twitter after the 2020-10-30 M7.0 Turkey earthquake (Test Dataset 2). The results for the real-time test on the Turkey earthquake are also good, with a recall of 77.7% and precision of 94.7% for damaged building class. Non-damage class recall is 94.4%, and precision is 98.9%. To understand what features in the images the model learns, we also implemented a Gradient-based localization method, Grad-CAM^17^, which can show us which part of the image the model is using to make the decision. By generating heatmaps using Grad-CAM, we can see the model correctly learned to find the damaged texture in the image to make the decision about damaged buildings.

## Methods

### Transfer learning with VGG19

**Figure 1:**
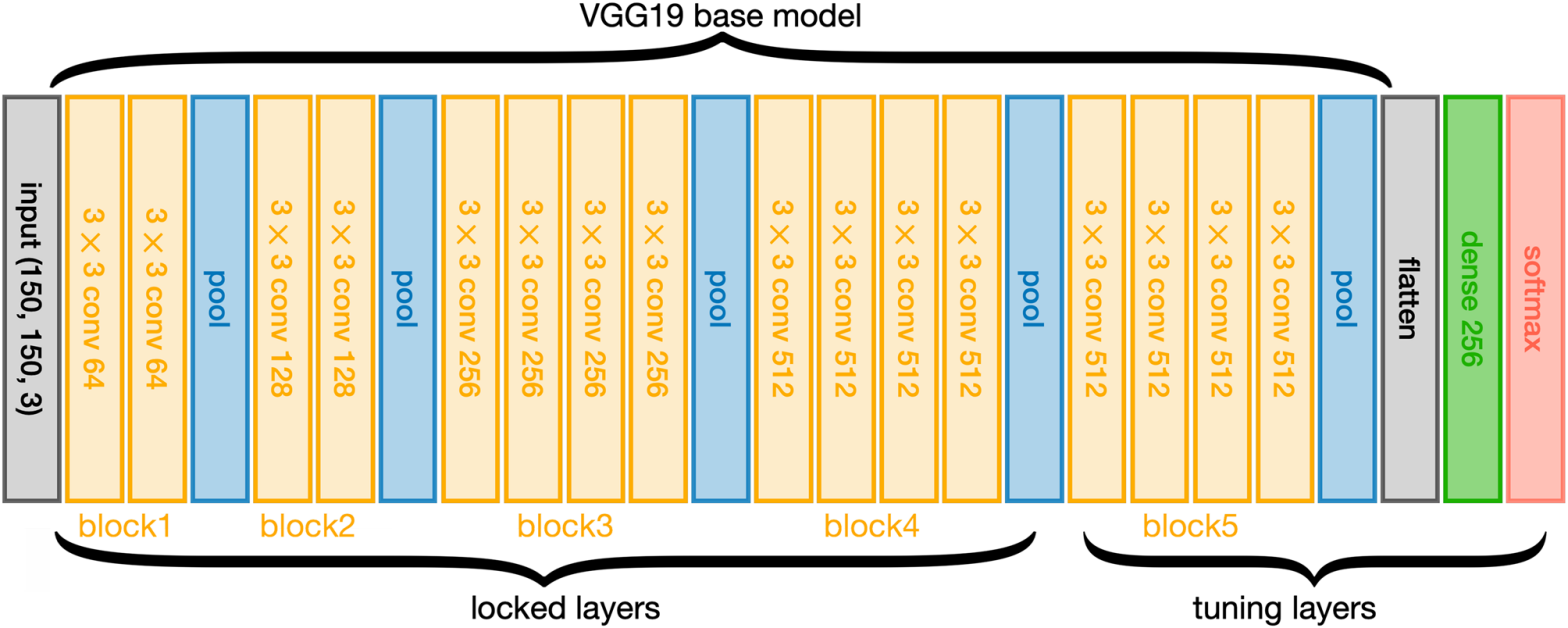
**Structure of the transfer learning model**. The input is (150, 150, 3), i.e. the image dimension is 150 by 150 pixels, with 3 channels. Convolutional layers are the yellow boxes with kernel size written in the front and number of kernels in the end; for example 3 × 3 conv 128 means kernel size is 3 by 3 pixels with a total of 128 kernels. Blue boxes are max pooling layers. Green box represents the fully connected dense layer with 256 neurons. Red box is the output layer with the softmax layer to output the classification results for the two classes.

We built our model on top of the VGG19 model, which was trained on the ImageNet dataset. Figure 1 shows the transfer learning architecture. The first 4 blocks of layers were locked and we only allowed the weights in the 4 convolutional layers in block 5 to vary to fine tune the model. After flattening the feature maps from block 5, a fully connected layer with 256 neurons was added before feeding into the softmax layer to make the final decision.

**Figure 2:**
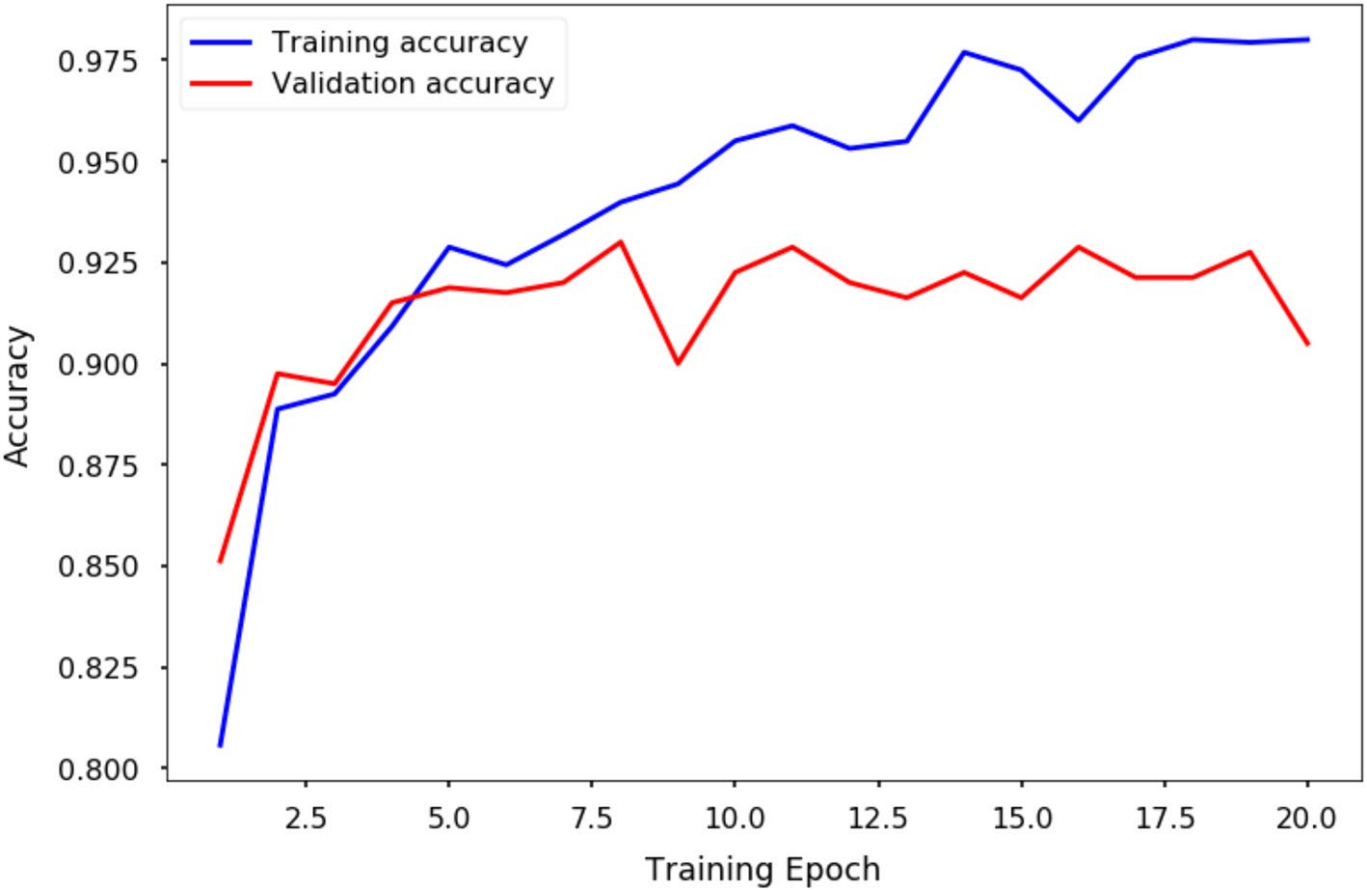
**Training accuracy curve**. Blue curve is the training accuracy while the red curve is the validation accuracy that was used for monitoring where to pick the best model that is not overfitting.

Input images were rescaled to 150 pixel by 150 pixel for all 3 channels and we normalized the values between 0 and 1. Due to the imbalance of the training datasets (3684 non-damage images and 2872 damaged building images), we use the class weights 0.8897 for non-damage class and 1.1414 for damaged building class to weight the loss function (during training only) to tell the model to "pay more attention" to samples from damaged building class. A categorical cross-entropy loss function was used with the RMSprop optimization algorithm (with initial learning rate set at 1e−5). The training accuracy curve is shown in Fig. 2, and training was conducted on one K80 GPU using Google Cloud Platform with each epoch taking about 20 s. The best model was achieved at epoch 8. For further details, refer to the Jupyter notebook on the provided github repository (see the Code Availability section).

### Baseline CNN model

In order to have a baseline performance for comparison, we also built a standard convolutional neural network to serve as a baseline model. Due to the relatively small labelled dataset, we only used up to 4 CNN layers in the model. The model structure and training details can be found in the supplementary material.

### Grad-CAM

To better understand what the model is relying on during the inference, we visualize the deep neural network we built via the gradient-based localization algorithm—Grad-CAM^17^. The basic idea of this method is that the last convolutional layer extracts the feature maps that have the best compromise between high-level semantics and detailed spatial information, and that the whole model will make the decision based on these feature maps. By taking the gradients of the class score with respect to the feature maps, they provide a good indication of the pixels that are important to the final decision. More specifically, using the symbols used in the Grad-CAM paper^17^, we can first take the gradient of the score ${y}^{c}$ for class c, with respect to the last convolutional layer feature maps ${A}^{k}$, where k is the ${k}^{th}$ feature map, and globally average them across all the pixels to obtain the importance weights ${\alpha }_{k}^{c}$ for each feature map, as shown in the Eq. (1):

1 $${\alpha }_{k}^{c}=\frac{1}{Z}\sum \limits_{i}^{u}\sum \limits_{j}^{v}\frac{\partial {y}^{c}}{\partial {A}_{ij}^{k}}$$

where u and v are the width and height of the feature map, and i and j are the (i, j) location on the feature map. These weights give the relative importance of each of the feature maps for the target class c before the model makes decisions. Then the class discriminative localization map Grad-CAM ${L}_{Grad-CAM}^{c}$ can be calculated using Eq. (2):

2 $${L}_{Grad-CAM}^{c}=ReLU({\sum }_{k}^{n}{\alpha }_{k}^{c}{A}^{k})$$

where n is the total number of feature maps in the last convolutional layer, and ReLU is the Rectified Linear Unit. The reason to apply a ReLU to the linear combination of the weighted feature maps is that only the features that have a positive influence on the target class are needed. The derived localization map is a non-negative weighted average of all the feature maps in the same dimension of the last feature map, and it is up-sampled to the input image resolution using bi-linear interpolation to form the final heatmap. This heatmap can show the importance of the different regions on the input image influencing the final output to the target class.

## Results

### Performance of the transfer learning model

**Figure 3:**
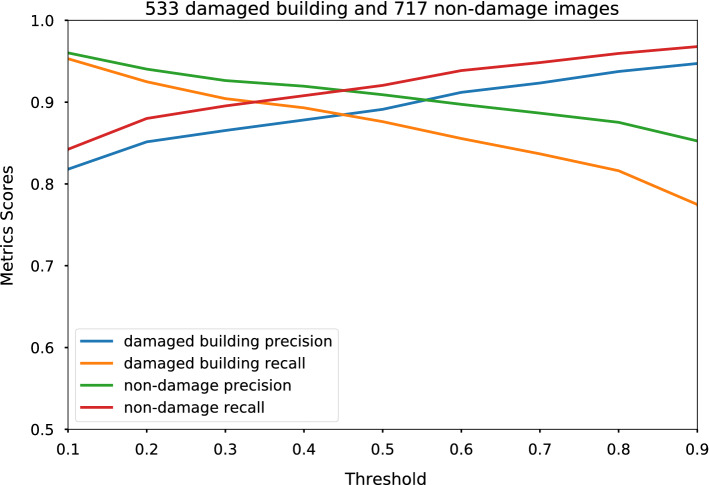
**Performance of the trained model on the validation dataset**. The horizontal axis shows the different decision thresholds, and the vertical axis shows the metrics scores for different metrics used. The four different colored lines are the precision and recall scores for damaged building and non-damage classes respectively.

The trained model (see the “Method” section for the details of the transfer learning) performs well on the reserved validation dataset, which consists of 533 damaged building images and 717 non-damage images. Figure 3 shows the precision and recall for both classes by using different thresholds on the validation dataset; we decided to use 0.5 as our final threshold. This gives us precision and recall for the damaged building class of 0.89 and 0.87 respectively, while the precision and recall for non-damage class are 0.91 and 0.92. Note the imbalanced nature of the dataset, i.e. there are more images of the non-damage class than the damaged building class. This will be especially true in real-world applications; therefore, we keep these precision and recall metrics calculated using the imbalanced dataset.

**Figure 4:**
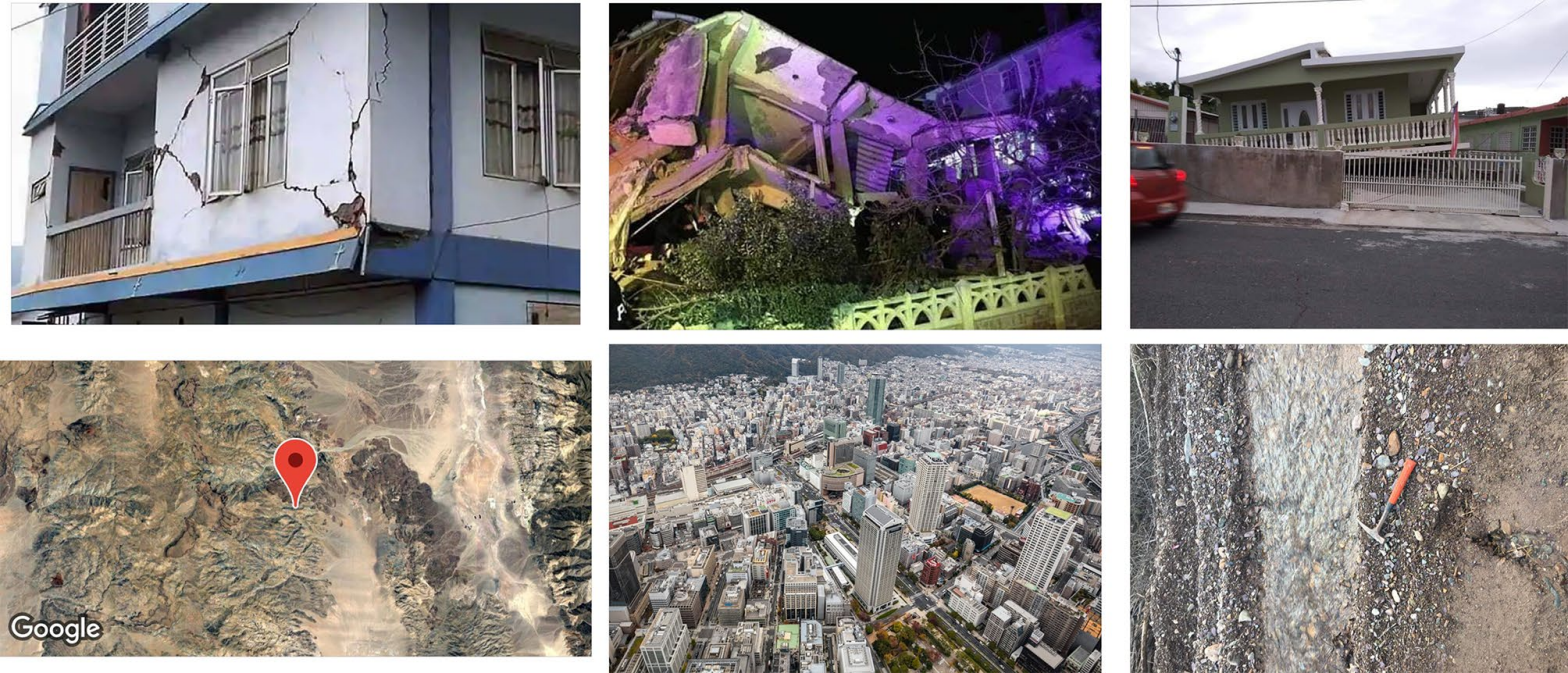
**Examples of images that cause the trained model to commit mistakes**. The upper row shows examples of damaged buildings erroneously classified as non-damage. From left to right: cracks in the building, night view of the building, and the whole building yielded or soft story collapse. The bottom row shows the non-damage images that were estimated as damaged building images. From left to right: Google satellite images, aerial view of the city and rock rubble similar to debris patterns.

In order to test the model performance, we downloaded a new test dataset from Twitter from the time period 2020-01-01 to 2020-09-03 (Test Dataset 1), which includes 24,058 non-damage images and 372 damaged images that are manually labelled by the authors. Table 1 shows the confusion matrix for this dataset; the corresponding damaged building recall is 80.5% and precision is 77.4%; non-damage recall is 99.6% and precision is 99.6%. A closer look at the images that are wrongly labelled enables us to identify areas where the model can be further improved. Of the 73 damaged building images that the model missed, three types of mistake are common (see Fig. 4 upper row): buildings with serious cracks (9 images), night views of the building (13 images), and the entire building ground floor collapsed (also known as soft story collapse; 18 images). These 3 types of errors account for 54.8% of all errors. Within the 88 images that the model mis-labelled as damaged buildings when they were not damaged, 73.9% come from three categories: 37 of them are maps with the majority of them from Google satellite images, 13 of them are aerial images that are taken from a far distance, and 15 images have features very similar to building debris, such as rock piles, tree leaves etc. The bottom row in Fig. 4. shows examples of such images.

**Table 1:** Confusion matrix for the twitter test dataset after removing duplicate images.

	Predicted damage	Predicted non-damage	Precision	Recall
True damage	301	73	0.774	0.805
True non-damage	88	20,342	0.996	0.996

We did further tests of the model performance during the 2020-10-30 M7.0 Turkey earthquake (USGS event id: us7000c7y0) in near real-time. We ran the model to monitor the incoming Twitter feed for about 30 hours searching images with keywords “earthquake”, and let the model classify these images. We then removed the duplicates from the images and manually labelled the images to compare the results with the automatically generated labels. The confusion matrix for the output results is shown in Table 2. The model performs quite well with a damage recall of 0.78, damage precision of 0.95, non-damage recall of 0.99, and non-damage precision of 0.94.

**Table 2:** Confusion matrix for the near-real time test on M7.0 Turkey Earthquake after removing duplicate images.

	Predicted damage	Predicted non-damage	Precision	Recall
True damage	160	46	0.947	0.777
True non-damage	9	777	0.989	0.944

Furthermore, we trained a 4-layer convolutional neural network (CNN) from scratch to serve as a baseline model for comparison purposes. The details of the baseline model is in supplementary material. We find that the performance of the baseline model is worse than that of the transfer learning. Using a threshold of 0.2, the damaged building precision is 40%, while the recall is 86.0%. If a threshold of 0.5 is used, then the damaged building precision is 51.1%, but the recall is 66.4%. Neither of these performances are close to the transfer learning performance.

### Visualizing the results using Grad-CAM

**Figure 5:**
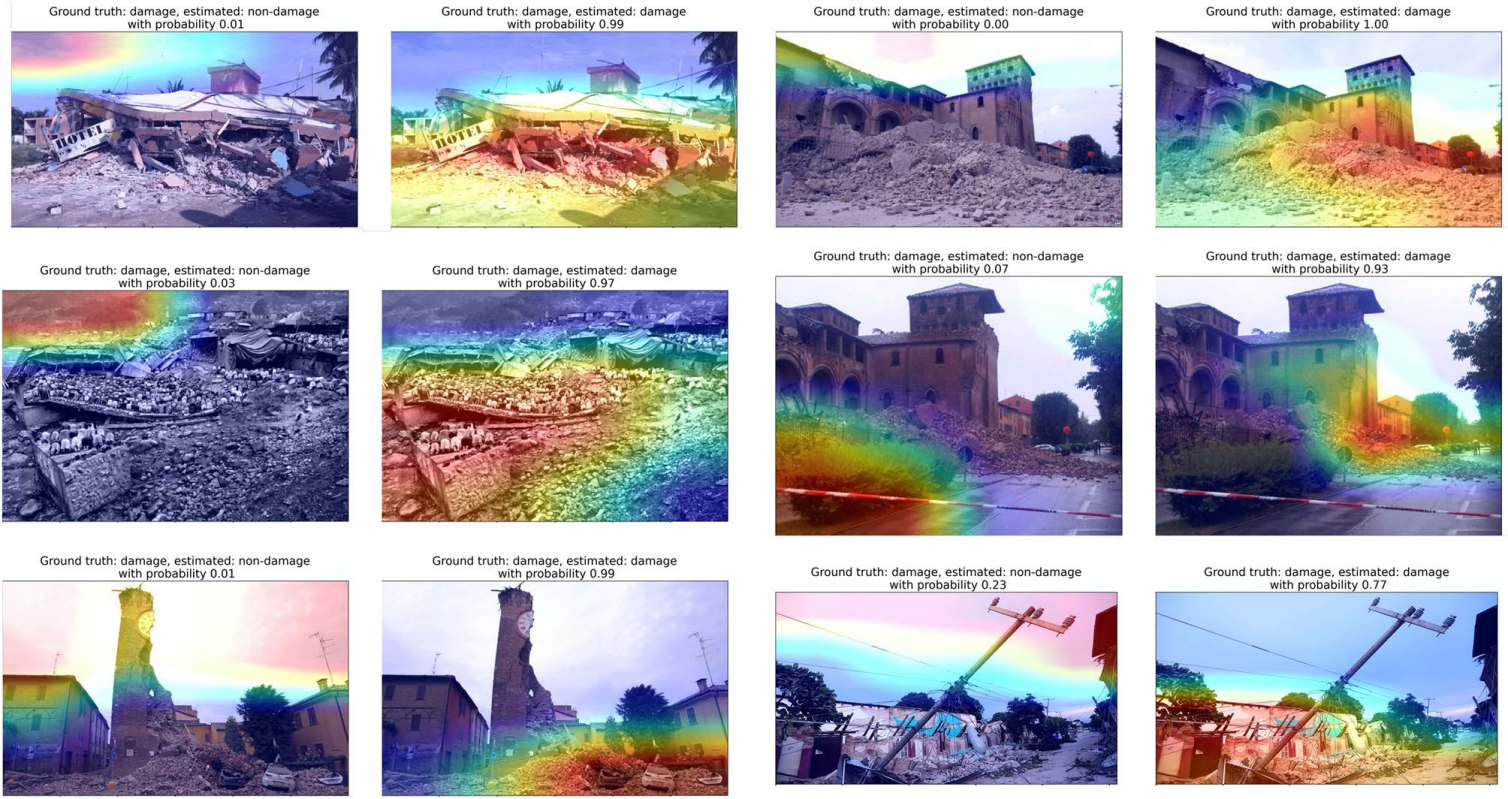
**Grad-CAM heatmap on images that the model made the correct decision for damage-building class (True Positives)**. Heatmap overlaying images indicates where the model pays more attention when making the decision; warmer colors indicate stronger influence on the decision. Each image is shown twice and overlaid with a heatmap indicating the regions of the image that suggest building damage, and the regions that suggest no building damage. The ground truth, estimated label and estimated probability is shown in the title above each image.

**Figure 6:**
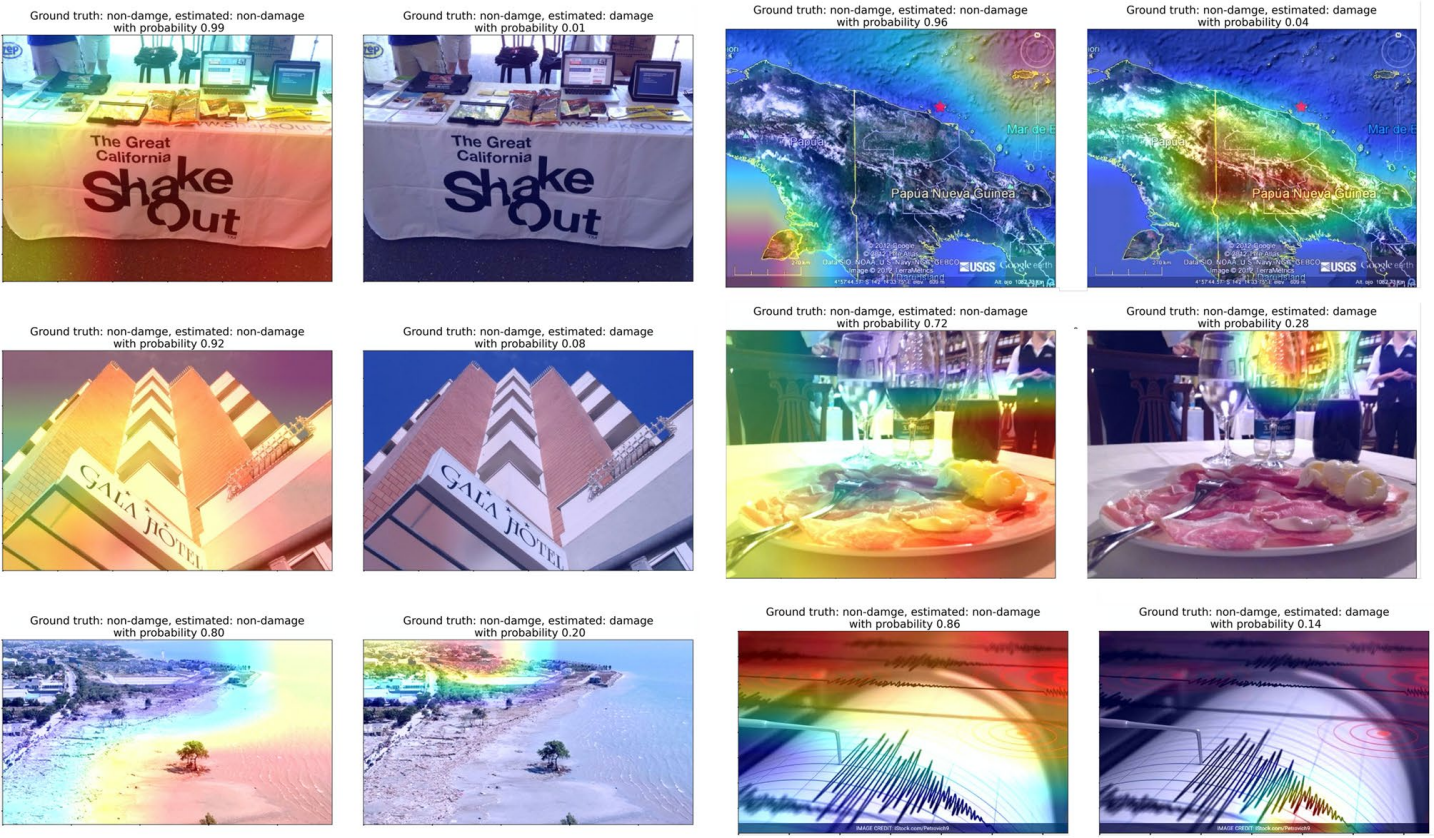
**Grad-CAM heatmap on images that the model made the correct decision for non-damage class (True Negatives)**. Heatmap overlaying images indicates where the model pays more attention when making the decision; warmer colors indicate stronger influence on the decision. Each image is shown twice and overlaid with a heatmap indicating the regions of the image that suggest building damage, and the regions that suggest no building damage. The ground truth, estimated label and estimated probability is shown in the title above each image.

**Figure 7:**
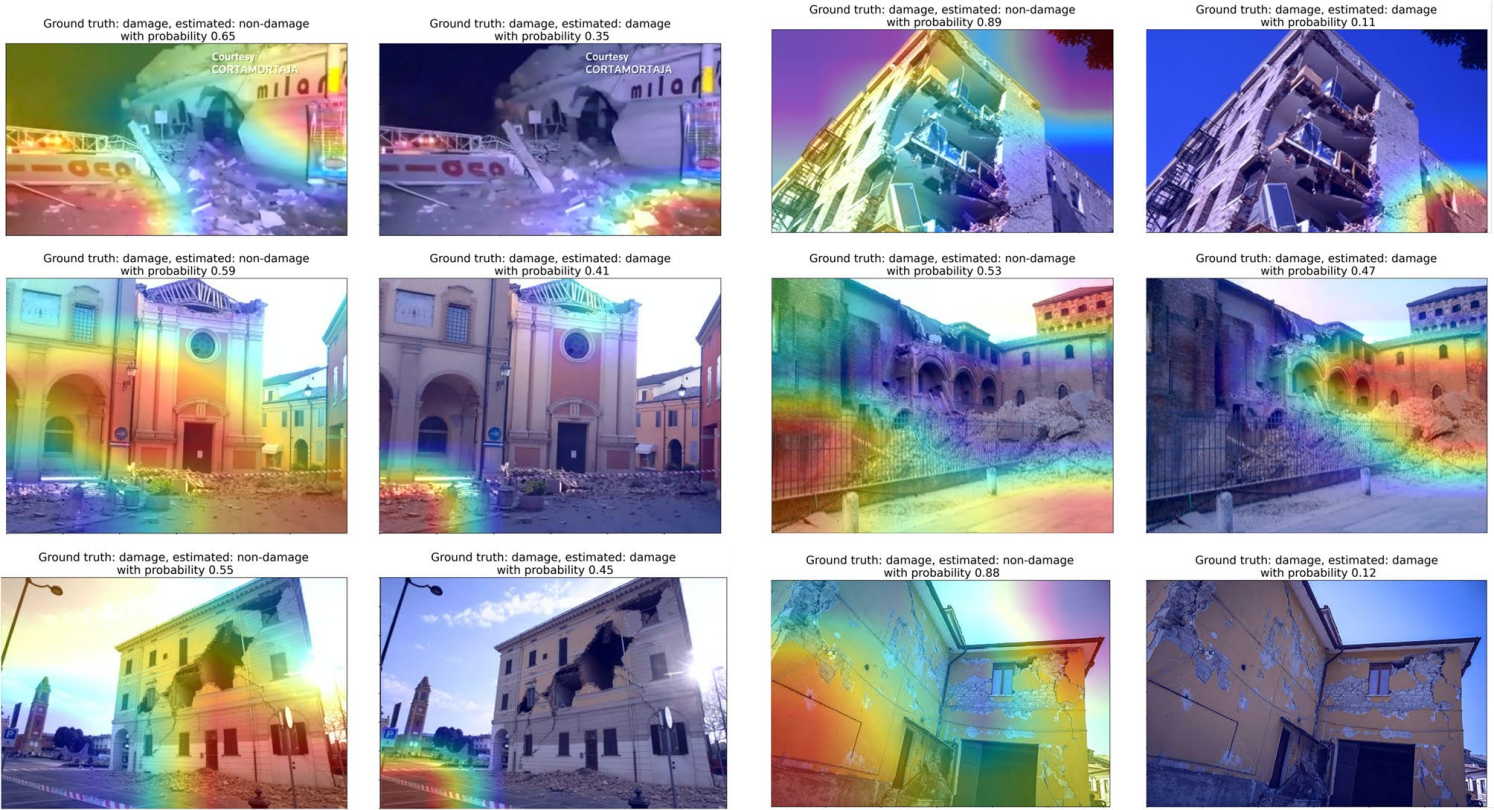
**Grad-CAM heatmap on images that the model made the wrong decision for damage-building class (False Negatives)**. Heatmap overlaying images indicates where the model pays more attention when making the decision; warmer colors indicate stronger influence on the decision. Each image is shown twice and overlaid with a heatmap indicating the regions of the image that suggest building damage, and the regions that suggest no building damage. The ground truth, estimated label and estimated probability is shown in the title above each image.

**Figure 8:**
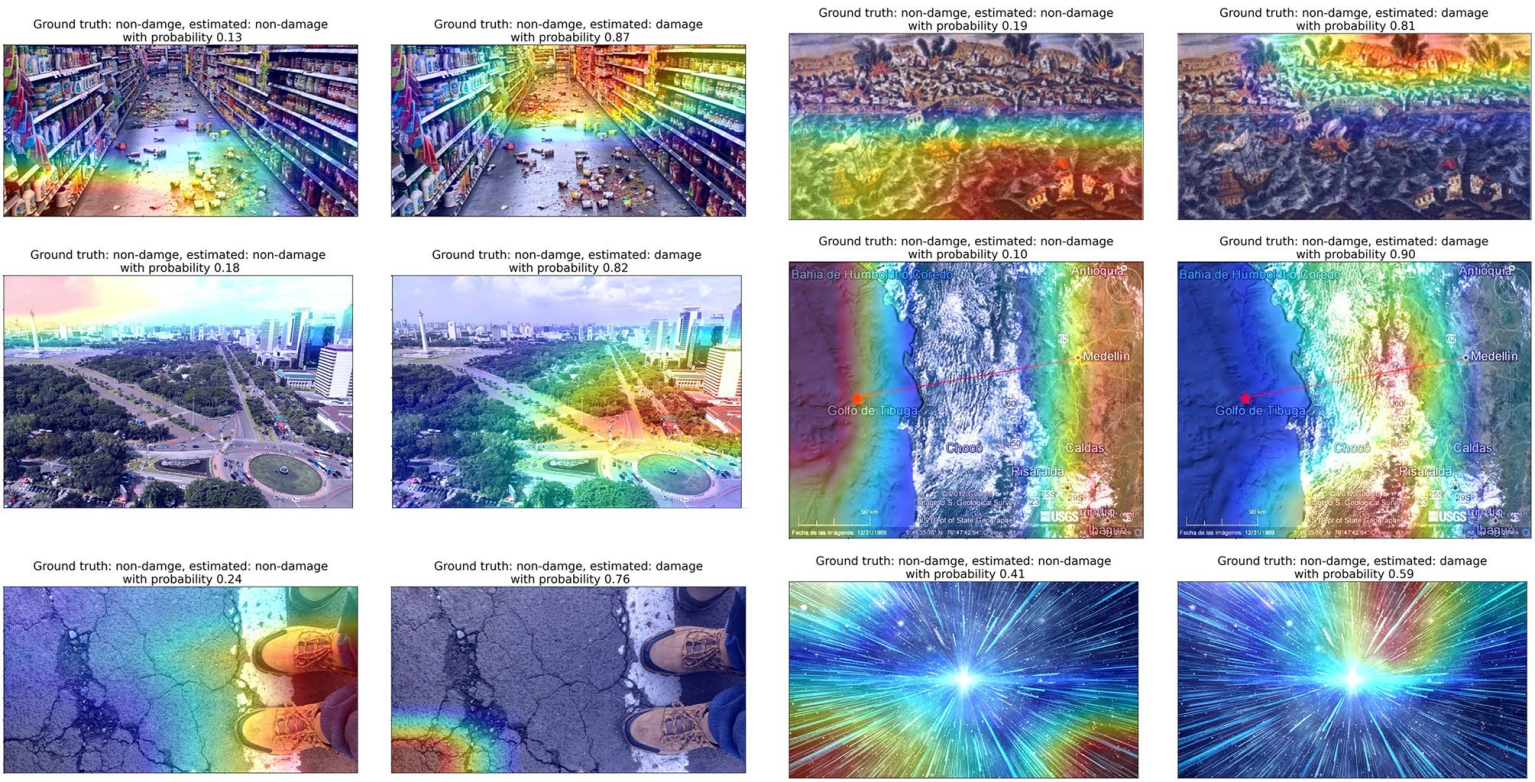
**Grad-CAM heatmap on images that the model made the wrong decision for non-damage class (False Positives)**. Heatmap overlaying images indicates where the model pays more attention when making the decision; warmer colors indicate stronger influence on the decision. Each image is shown twice and overlaid with a heatmap indicating the regions of the image that suggest building damage, and the regions that suggest no building damage. The ground truth, estimated label and estimated probability is shown in the title above each image.

We implemented the gradient-based localization algorithm—Grad-CAM to help us understand what aspects of the images the model was using to make the decision. Figures 5, 6, 7, 8 show the results of overlaying the Grad-CAM heatmap on the original image for both classes estimations.

We show the heatmaps for both classes to have a better view of what the model was focusing on to make the decision. For example, Fig. 5 top left panel shows building damages. The left image in the panel shows where the model focuses if it thinks the image is a non-damage class. Clearly, the model focuses on the sky part of the image, which is correct. The other panel shows where the model focused when looking for evidence of building damage. Overall, the probability that this image is a non-damage class is 0.01, which means the model made a correct decision that this is a damaged building. There is a tendency that the more features of the damaged buildings in an image, the higher is the confidence of the model, which can be seen from other panels in the figure. The model learns to identify the debris of the buildings very well; debris piles are often the feature that is the focus of the model in the images. However, other features that are similar to debris, such as grass fields and tree leaves, are occasionally mistreated as debris from damage. Examples of this are in Figs. 4 and 8. Figure 6 shows the true negatives, which the model correctly recognizes as the images without damaged buildings. From the focused features in these images, we can notice that in most instances the trained model focuses on the correct features to make the decision.

Figures 7 and 8 show the false negative and false positive cases. We can notice which features the model focused on when making wrong decisions. For instance, when there are damaged buildings, but the building is not the main focus of the image, the image is often classified as non-damage. This also occurs when there are other objects in the foreground, or other objects occupy a large fraction of the image, or the buildings are in dark (Fig. 7). Also, less catastrophic damage such as cracks in the buildings do not lead to a damaged-building label. This is likely due to the fact that only severely damaged buildings are captured by the model, as the majority of the training data contains only the severely damaged buildings. Figure 8 shows where the model tends to make false positive estimates, i.e. treat the non-damage features as damage. As mentioned earlier, features that are similar to debris are usually determined as damage features by the model.

## Discussion

We designed an end-to-end workflow using the transfer learning approach to extract images containing damaged buildings from social media platforms after earthquake disasters. The purpose of this study is to train a model that can identify the images with damaged buildings that users may upload to the MyShake app in the future after an earthquake. But the same method can be expanded broadly to extract images from social media platforms after a disaster. The implemented Grad-CAM method can help us to understand the learned features of the model and also diagnose potential improvements.

There are three main benefits of the pipeline we built here. Firstly, good performance can be obtained from a relatively small labelled dataset, thanks to the success of transfer learning developed in recent years. This can be used in many applications where collecting large amounts of labelled data is time consuming and costly. Here, we only used a few thousand labelled images to obtain a good working initial model. In addition, the computation gets dramatically reduced using transfer learning as opposed to building a model from scratch. Training takes about 20 s for each epoch on one K80 GPU, and with less than 20 epochs the transfer learning can converge to a good solution due to the smaller sets of trainable parameters. On the other hand, training a whole baseline CNN model from scratch needs about ~ 1.5 h to converge on the same GPU even though it has a much simpler structure. It is also notable that the performance of the baseline model trained from scratch on this small dataset is much inferior to that of the transfer learning model.

Secondly, the Grad-CAM method also shows whether the trained model focuses on what we expect. We can see in Grad-CAM overlaid images that the current trained model can focus on the obvious damage of the building correctly. However, we can notice from the false positives that the model confuses patterns like debris, such as tree leaves, grass fields etc. with the damaged building features. We find 73.9% images in the false positives are due to the features in the images that look like debris, either from aerial images, satellite maps or tree leaves. This is an area that we can focus on to make improvements in the future by providing more training data that contain this type of patterns.

Finally, while the current model is not perfect, it is a good initial model that we can use to filter a majority of images. A challenge that still remains is that there are many more images of the non-damage class than damaged buildings. A semi-automatic approach can be to use this model to initially filter a majority of images, and then manually filter out the false positives to further improve the model.

As we can see from the performance section, the model can be improved in a few places. Firstly, the false positives of the damaged buildings mainly come from the patterns that are similar to debris, such as the rock rubbles and tree leaves. In addition, satellite maps and aerial images can also trick the model, as some of these textures look like damaged building materials. One potential solution for this problem is to collect more images of these kinds and add them into the training dataset to make the model more robust for these features. Fortunately, such images are abundant on social media platforms and easy to collect compared with the damaged building images. Therefore, this could be the next step to improve the model.

Secondly, for the false negatives of the damaged buildings, such as the night scenes, and cracks in buildings, we may need a different approach other than collecting more training data, since such images may not be common on the social media platforms. For the night scene images, due to the low light source during the time of taking the pictures, the building objects in these images are usually blurry and dim, making it intrinsically hard to estimate the class. One way to get more of such night time images with damaged buildings is to artificially convert the day time images into night time images using image processing packages. This data augmentation step can theoretically improve the training dataset by turning the images taken in day time after the earthquakes to night time. On the other hand, there are also many research developments of enhancing the night scene images for object detection or even converting the night scene images to the day time images^18–21^. This approach could potentially make these night scene images easier for the model to assess. As for the images with just cracks in the buildings, they can be considered of low consequence. The purpose of this project is to detect severely damaged buildings after the earthquake. Still, if we aim to capture these cracks related to smaller earthquakes in the future, we can reach out to the civil engineering community for more training data as they also collect and develop machine learning models to identify building cracks after the disaster^22–25^. The soft story building collapse is another type of failure for which we need to make improvements. One way is to do more data argumentation (such as shift, rotate, increase contrast, brightness etc.) on these types of images to increase the number of training cases in the current training datasets. However, reaching out to the civil engineering community for more training image data may be more effective.

As stated in the motivation of this study, we hope that in the future MyShake users can upload images they take after an earthquake to report building damage. These images could be displayed on the map in the app to better inform users about damage in their neighborhood. The current designed model only performs binary classification to indicate whether the image contains damaged buildings or not. As more databases of these images are accumulated overtime, we could also assign a score for the severity of the damage in each image, and build another machine learning model to estimate the severity of the damage in each image that contains damaged buildings. We hope that the proposed model can be used as a tool to collect damage related images for the earthquake engineering community for further research applications, such as damage quantification and rescue prioritization.

### Training dataset

For this work, we decided to use training images from earthquake events at two locations: Nepal and Indonesia. We then test the trained model on other locations/events to assess its performance. We began the data collection with Twitter as the source since high social media activity occurs on this platform following earthquakes and such activity on Twitter is predominantly open for public access.

A historical search of the Twitter feed was conducted for the 2015 Nepal earthquake event. An advanced search was conducted using GetOldTweets3 python library (https://pypi.org/project/GetOldTweets3/) and tweet_id were extracted from the results. The following search parameters were used: Keyword: earthquake; Filter: images; Date Range: April 25, 2015 to April 30, 2015.

Subsequently, the Twython python library (https://twython.readthedocs.io/en/latest/) was used to fetch the url of each image attached to every tweet_id. It is quite frequent on Twitter that many tweets can reference the same image. Thus, we only fetched images with unique urls. A total of 47,785 images were obtained from this search, which contained pictures of damaged buildings, other damaged structures (e.g. roads, bridges, etc.), and a wide variety of other pictures related and unrelated to the earthquake event. A number of cleaning operations were performed on this dataset and are described as follows: firstly, we found that even though we fetched images with only unique urls, many of them were still duplicates, which was assessed by comparing md5 hash of the images. All such duplicates were removed from the dataset. Secondly, we chose to remove images that were smaller than 150 px in either height or width. Thirdly, we found that there were many images that were even though not exact duplicates, they represented the same shot but differed in either just pan or zoom. Such images were processed through a proprietary software, Duplicate Photo Cleaner. This software allowed us to identify images that are similar by specifying a user chosen extent of similarity (in percentage). In this study, we chose a threshold of 50% similarity and only 1 copy from each set of images identified as “similar” was retained and the rest were removed. The total number of images thus obtained from Twitter post these operations was reduced from 47,785 to 18,238. A manual operation was performed to label a subset of the obtained images in two classes, viz. damage and non-damage.

**Figure 9:**
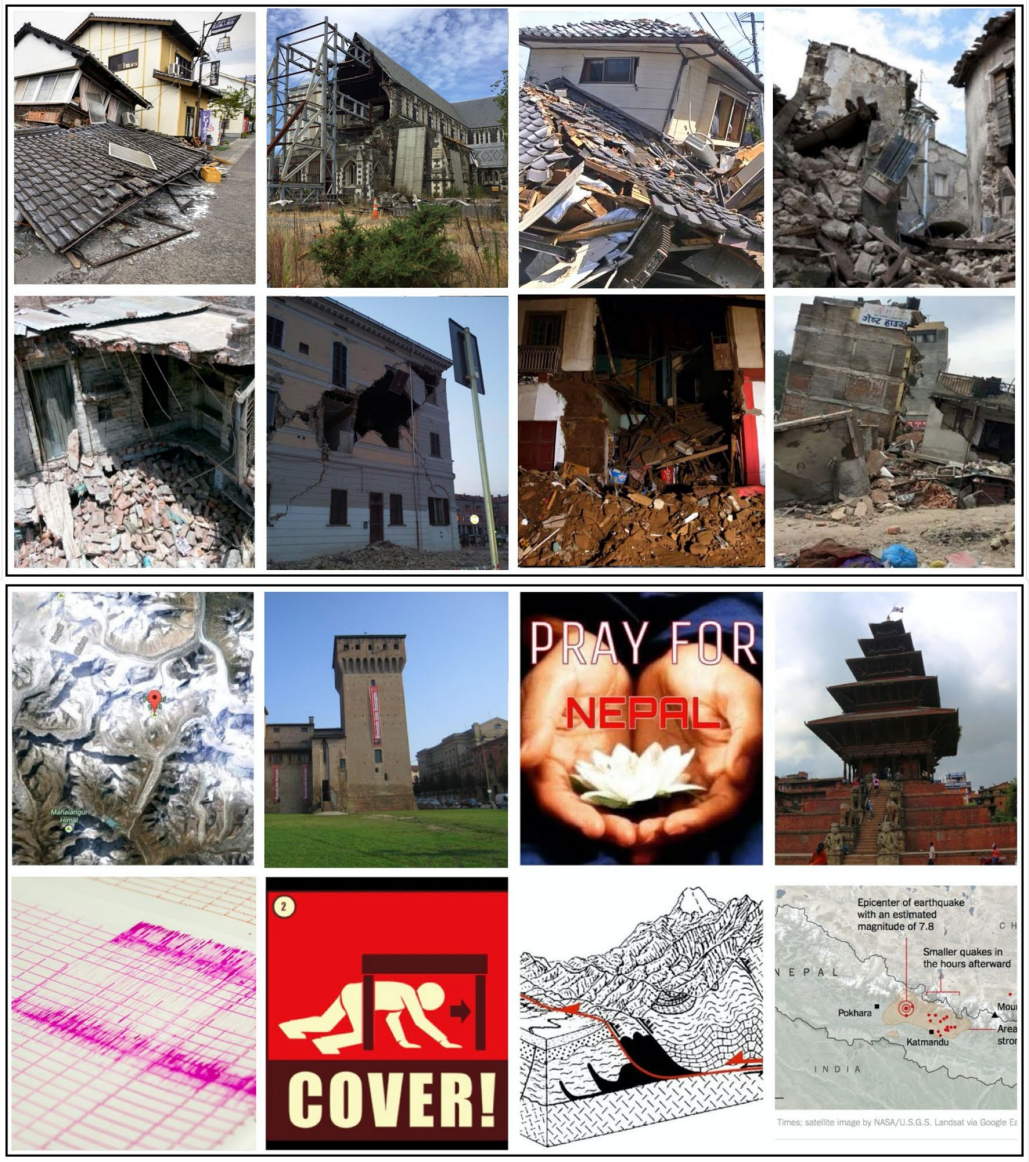
**Training data examples**. The upper panel 8 images are labelled damaged buildings, while the lower panel 8 images are labelled non-damage. All images are from Twitter.

An image was labelled damage when more than ~ 20% of the image area contained visible damage to a building structure in the form of debris, rubble, cracks, etc. See the upper 8 images in Fig. 9 for examples. The non-damage label was applied to any image which either contained buildings that were not damaged and/or contained completely unrelated pictures, like a portrait of a person, text, map, etc. See the lower panel 8 images in Fig. 9 for a few examples.

In all, 2337 images were manually labelled out of which 367 were classified as damage while 1970 as non-damage images. Thus, only about 16% images were obtained of the damage class. In order to obtain images of damage classification with better efficiency, we explored Getty Images as another data source. Publicly available images on Getty Images platform (https://www.gettyimages.com/) were obtained using an advanced search. We used “earthquake destruction” as our search term while setting the location to Nepal and Indonesia individually for these two places.

This resulted in 4219 images from Getty Images which were subsequently labelled manually. 2505 images were labelled as damage class while 1714 images were labelled as non-damage class. A csv file of the web links to these images made publicly available by Getty Images for free is included in Supplementary Material.

The two sets of data (from Twitter and Getty Images) were combined to form the training dataset of a total of 6556 images, comprising 2872 damage class and 3684 non-damage class. A schematic on this data collection process is shown in Fig. S4 in the supplementary material. The links to these images are stored in Tables S3 and S4.

### Excluded images

Certain images were found to not meet either of the criteria above and were excluded from the training dataset. Figures S5 and S6 in the supplementary material illustrates a few examples of such images. They essentially fall in two categories: (1) The damaged structure is not a building. Such images may be of damaged roads, vehicles, building interiors, etc. (2) The picture consists of elements other than the original shot. This included overlaying text on the image, screenshot of a news clipping, collage of different images, etc.

### Validation and test dataset

#### Validation dataset

The validation dataset was prepared with an aim to tune the threshold. These images were collected from Getty Images with “earthquake destruction” as our search term with locations set as “Italy” and “Mexico”.

1250 images were obtained from Getty Images and subsequently were labelled manually. 533 images were labelled as damage class while 717 images were labelled as non-damage class. A schematic on this data collection process is shown in Fig. S7. Links to these images are stored in Tables S5 and S6.

#### Test Dataset 1

A historical search of the Twitter feed was conducted for any earthquake related tweets between Jan 1, 2020 and September 30, 2020. An advanced search was conducted by employing the similar approach as for the training dataset, using the python libraries GetOldTweets3 (https://pypi.org/project/GetOldTweets3/) and Twython (https://twython.readthedocs.io/en/latest/). The following search parameters were used: Keyword: earthquake; Filter: images; Date Range: Jan 1, 2020 to September 30, 2020. A total of 24,640 images were obtained. After removing the duplicated images, we have 20,804 images remaining. Links to these images are stored in Tables S7 and S8.

#### Test Dataset 2

The second test dataset was prepared with the aim of running the model in near real-time immediately after a major earthquake event. During the course of this work, an earthquake occurred in Turkey on October 30, 2020 (2020-1030 M7.0 Turkey earthquake). A live stream of the twitter feed was analyzed to capture Twitter activity related to the earthquake. The live Twitter feed was processed for a total of 29 hours and 45 minutes between 10/30/2020 21:15:00 PST and 11/1/2020 20:22:00 PST using the keyword “earthquake” and filter “images”. Due to some connectivity issues during the live stream the connection had to be reestablished a few times, thus reducing the total number of active hours of data retrieval. A total of 992 images were obtained after removing the duplicates. Links to these images are stored in Tables S9 and S10.

## Supplementary Information

Supplementary Information 1.

Supplementary Table S3.

Supplementary Table S4.

Supplementary Table S5.

Supplementary Table S6.

Supplementary Table S7.

Supplementary Table S8.

Supplementary Table S9.

Supplementary Table S10.

## Data Availability

All the data described below are listed as the image ids in the csv files in the supplementary materials. Please note that due to deletion from the owners of the original post/image, a small portion of the links may not work. All the images shown in this paper are from Twitter which are subject to the policy of free use in Non-commercial usage: https://developer.twitter.com/en/developer-terms/agreement-and-policy. The Getty images used in this paper are only downloaded from the website for training purposes, and have been deleted after training. This follows the requirements from Getty Images “You are welcome to use content from the Getty Images site on a complimentary basis for test or sample (composite or comp) use only, for up to 30 days following download. However, unless a license is purchased, content cannot be used in any final materials or any publicly available materials. No other rights or warranties are granted for comp use.” from https://www.gettyimages.com/eula.

## Code availability

The code for the demo can be found in Github repository: https://github.com/qingkaikong/damaged_building_detection.
