# Supplementary material for: Detecting damaged buildings using real-time crowdsourced images and transfer learning
Source: Sci Rep. 2022 May 27;12:8968. doi: 10.1038/s41598-022-12965-0 (PMC9142599; doi:10.1038/s41598-022-12965-0)
Supplement: Supplementary file 1 — Supplementary Information 1. [file 41598_2022_12965_MOESM1_ESM.docx]

**Supplementary Information for Detecting Damaged Buildings Using Real-time Crowdsourced Images and Transfer Learning**

Gaurav Chachra, Qingkai Kong, Jim Huang, Srujay Korlakunta, Jennifer Grannen, Alexander Robson, Richard Allen

# Supplementary Figures


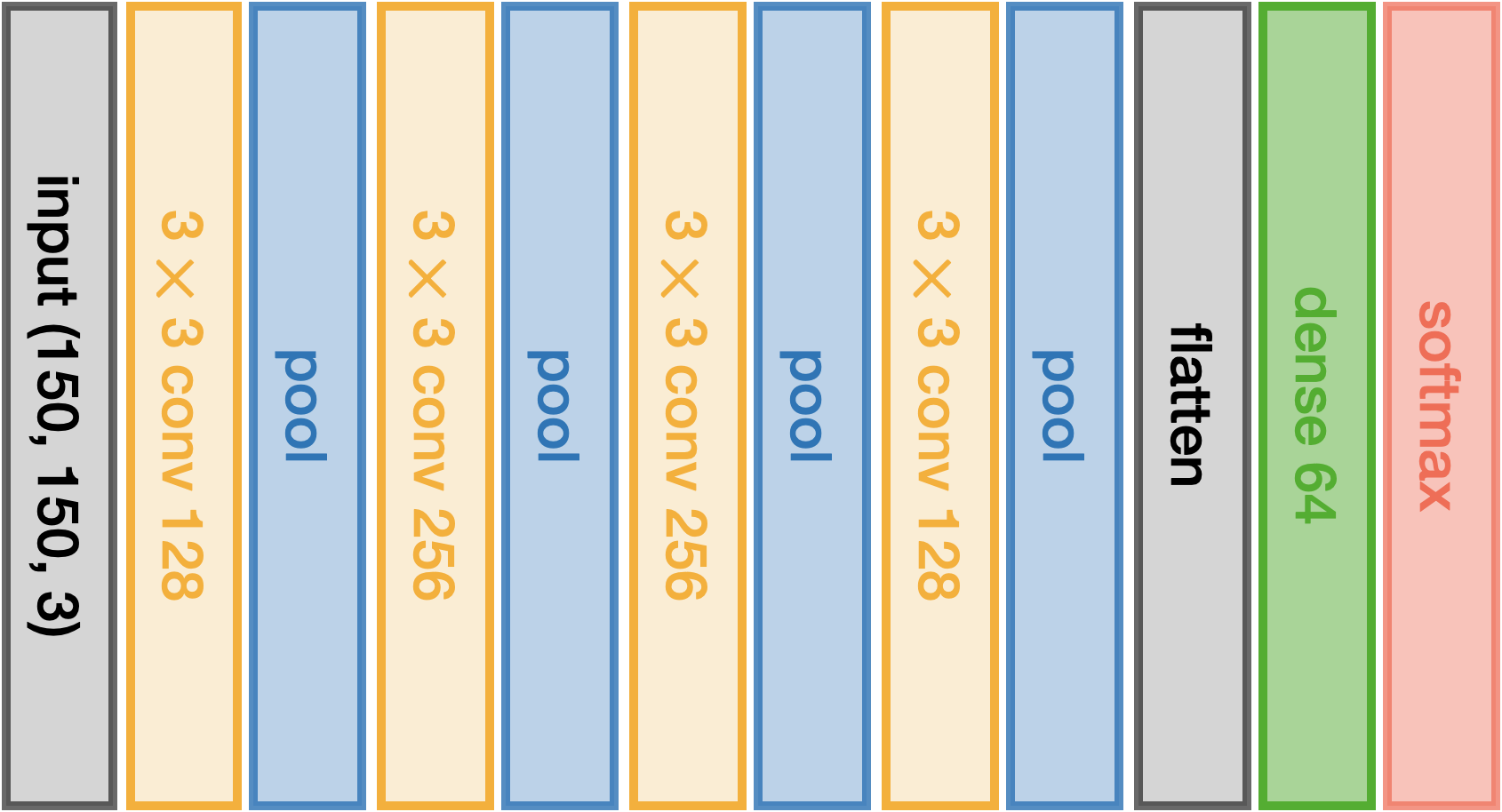


Fig. S1 - The structure of the baseline CNN model. Different colors of the blocks represent the different layers. The input is (150, 150, 3) meaning the image dimension is 150 by 150 pixels, with 3 channels. Convolutional layers are the yellow boxes with kernel size written in the front and number of kernels in the end, for example 3 x 3 conv 128 means kernel size is 3 by 3 pixels with a total of 128 kernels. Blue boxes are max pooling layers. Green box represents the fully connected dense layer with 64 neurons. Red box is the output layer with the softmax layer to output the classification results for the two classes.


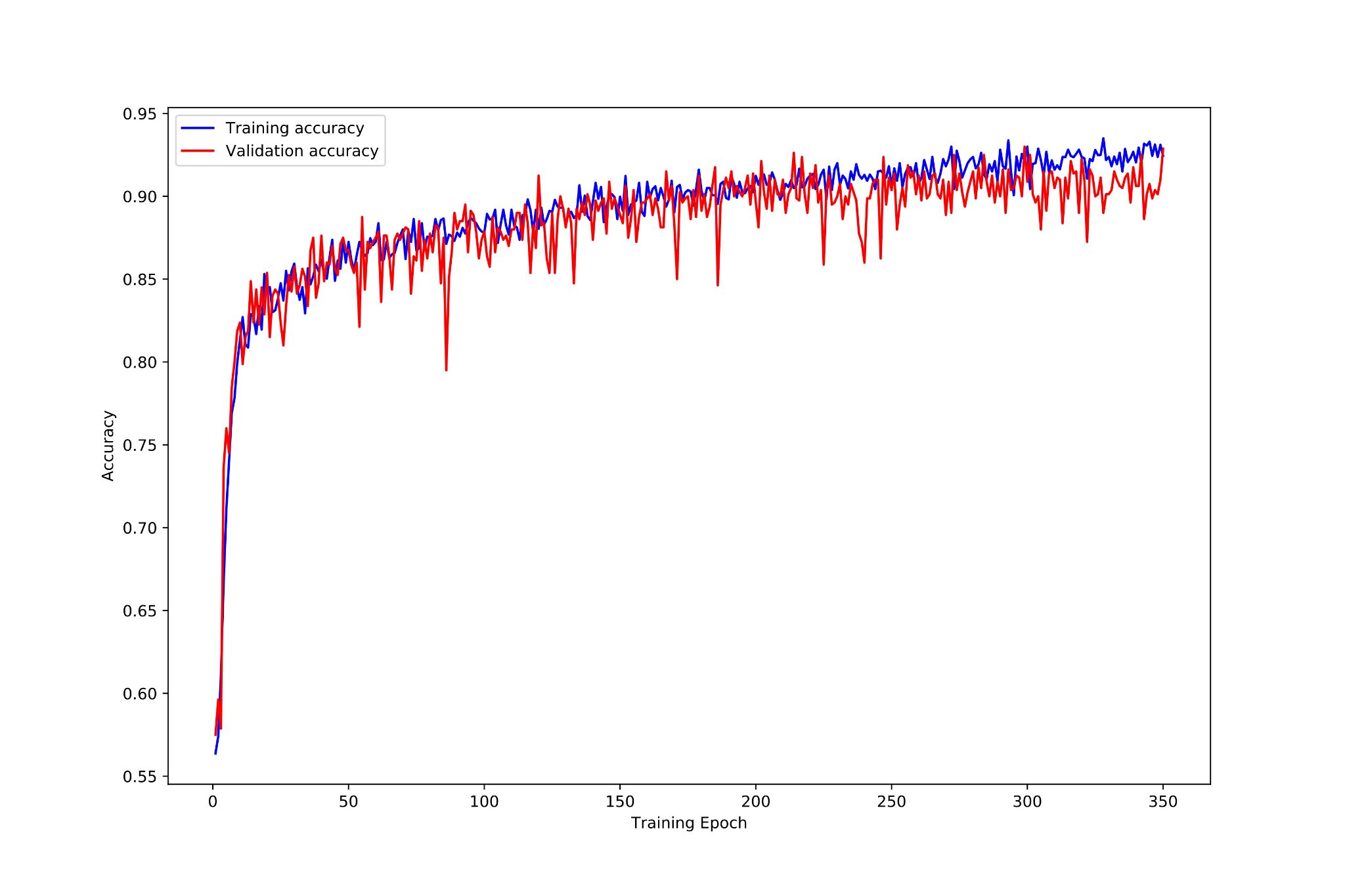


Fig. S2 - The training curve for the baseline CNN model.


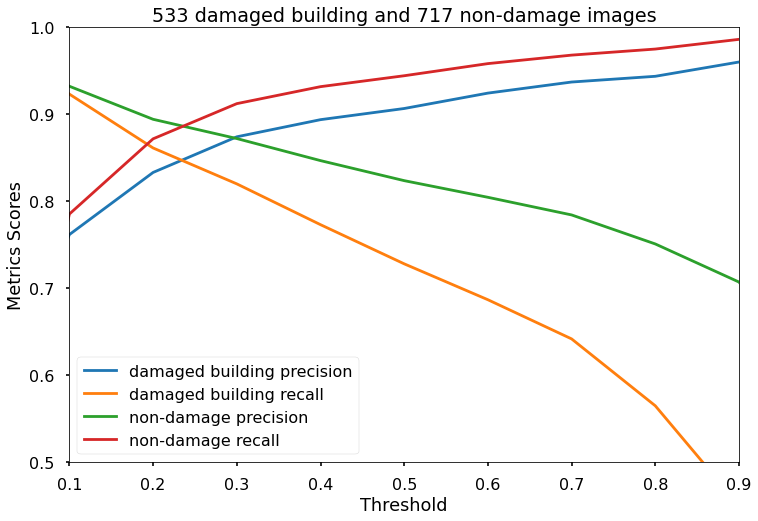


Fig. S3 - The validation dataset performance.


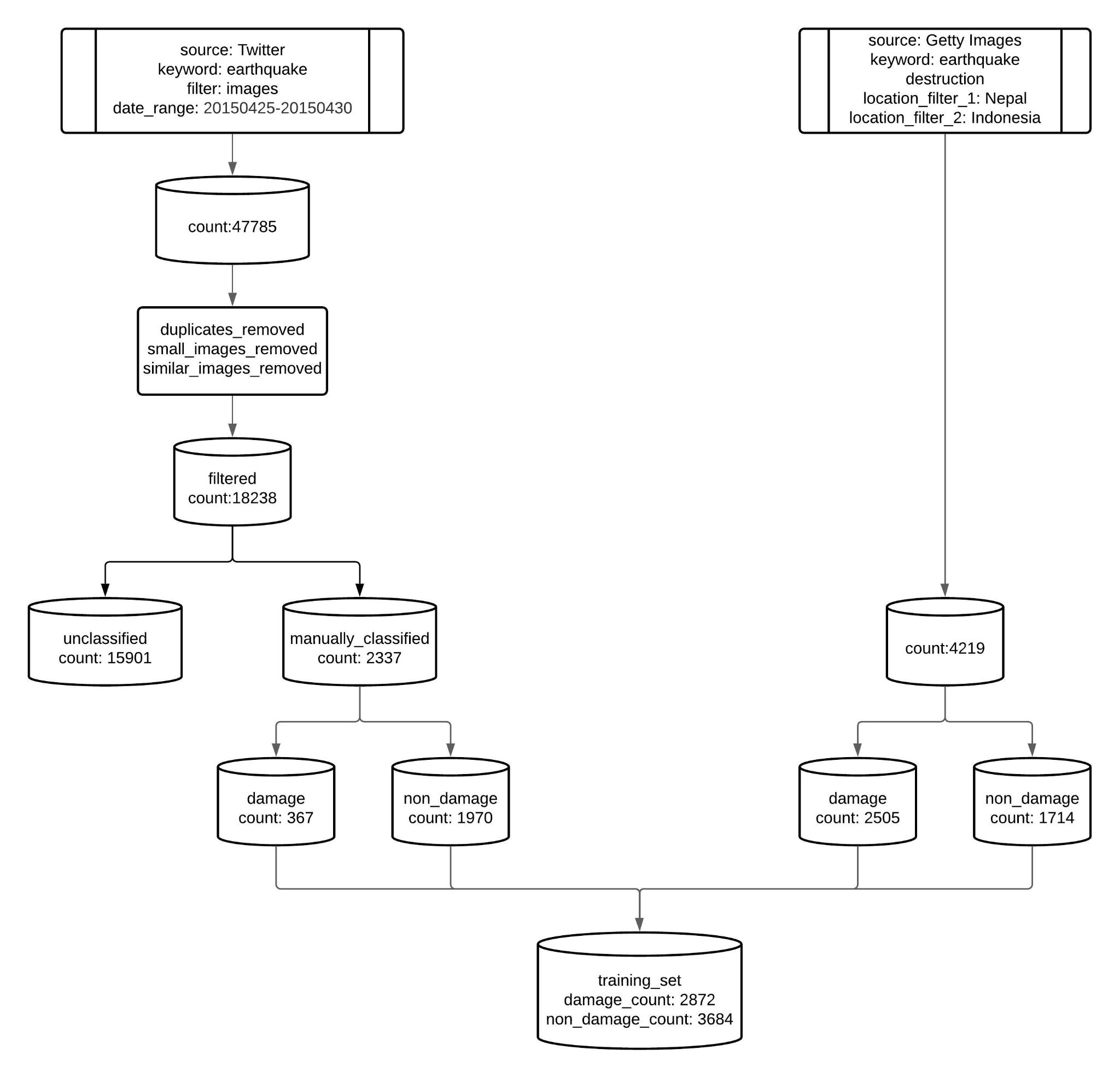


Fig. S4 - The schematic showing the process of collecting the training dataset.


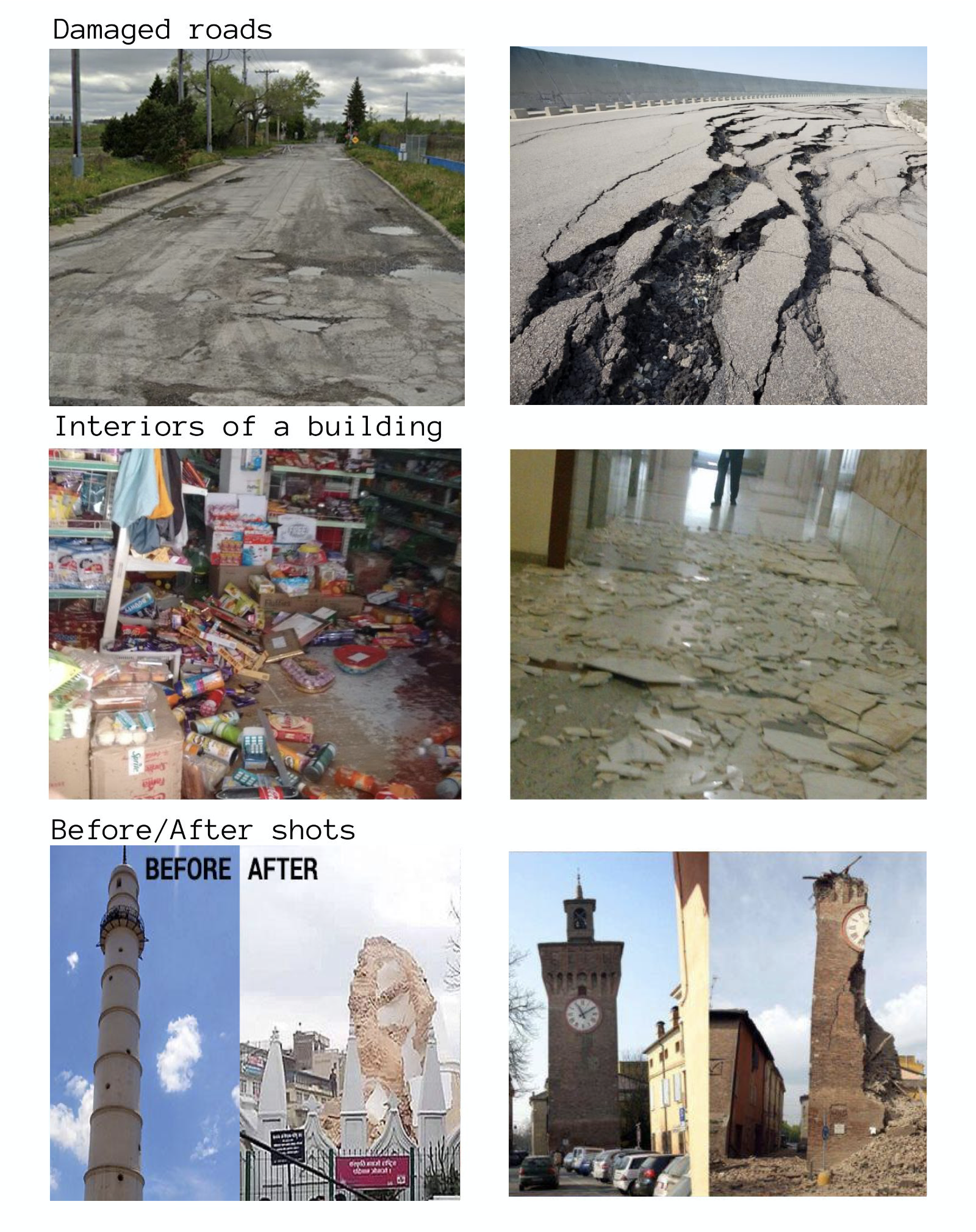


Fig. S5 - Images excluded from training dataset.


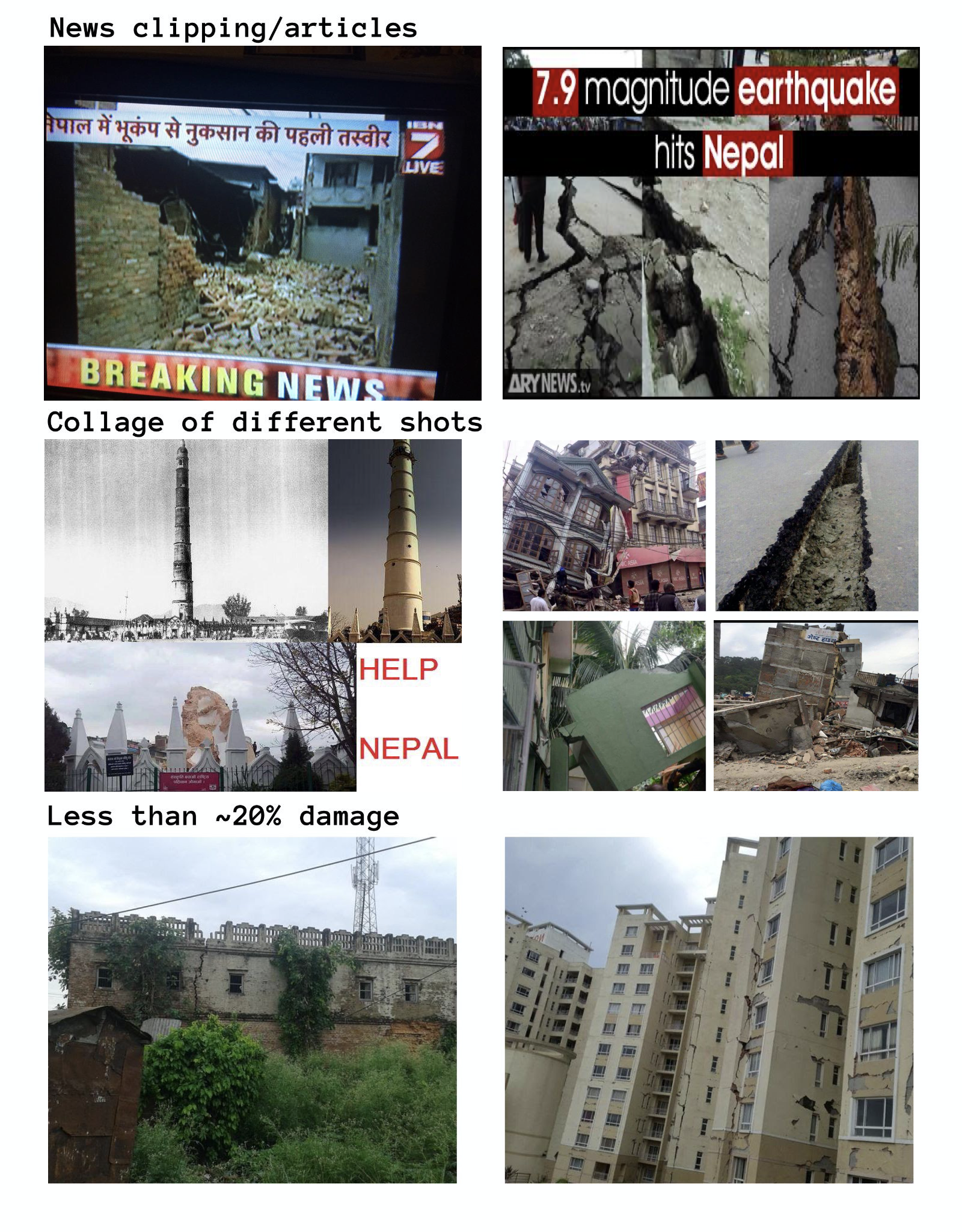


Fig. S6 - Images excluded from training dataset.


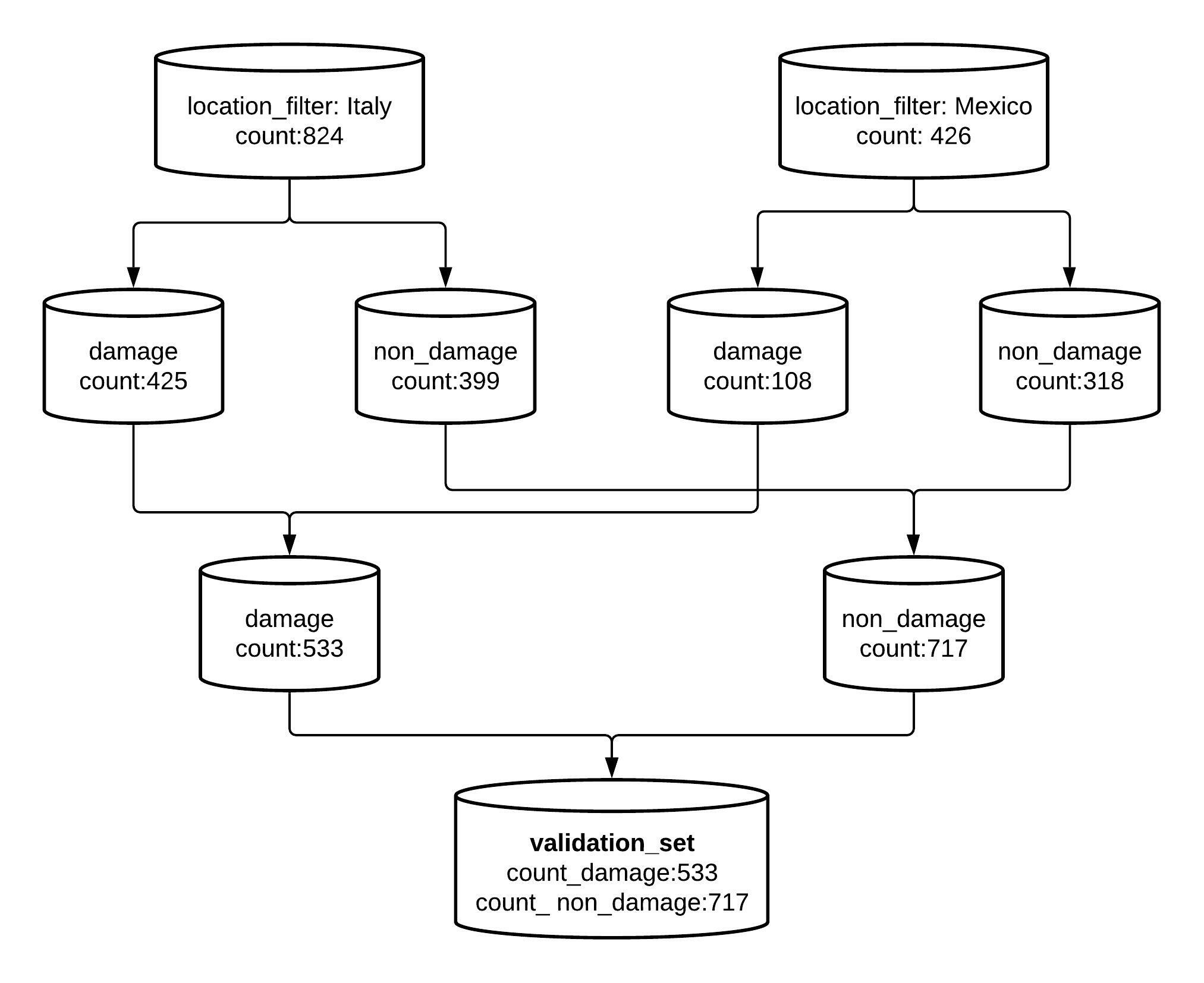


Fig. S7 - The schematic showing the process of collecting the validation dataset.

# Supplementary Tables

# **Table S1 Confusion matrix for the baseline model using threshold 0.2**

|  | **Predicted damage** | **Predicted non-damage** |
| --- | --- | --- |
| True damage | 320 | 52 |
| True non-damage | 472 | 23586 |

# **Table S2 Confusion matrix for the baseline model using threshold 0.5**

|  | **Predicted damage** | **Predicted non-damage** |
| --- | --- | --- |
| True damage | 247 | 125 |
| True non-damage | 236 | 23822 |

Table S3 - links to the damage-class images used in training dataset.

Table S4 - links to the non-damage-class images used in training dataset.

Table S5 - links to the damage-class images used in validation dataset.

Table S6 - links to the non-damage-class images used in validation dataset.

Table S7 - links to the damage-class images used in test 1 dataset, i.e. from tweets between Jan 1, 2020 and September 30, 2020.

Table S8 - links to the non-damage-class images used in test 1 dataset, i.e. from tweets between Jan 1, 2020 and September 30, 2020.

Table S9 - links to the damage-class images used in test 2 dataset, i.e. from live Twitter feeds from 10/30/2020 21:15:00 PST to 11/1/2020 20:22:00 PST.

Table S10 - links to the non-damage-class images used in test 2 dataset, i.e. from live Twitter feeds from 10/30/2020 21:15:00 PST to 11/1/2020 20:22:00 PST.
